# Correction: A case of afterload mismatch associated with shivering leading to fatal hypoxia in a COVID-19 patient

**DOI:** 10.1186/s40981-022-00548-x

**Published:** 2022-08-04

**Authors:** Takanori Suzuka, Yusuke Naito, Keiko Uemura, Mitsuru Ida, Junji Egawa, Masahiko Kawaguchi

**Affiliations:** Department of Anesthesiology, Nara Medical University, Kashihara, Japan

**Correction: JA Clin Rep 8, 51 (2022)**

**https://doi.org/10.1186/s40981-022-00542-3**

Following the publication of the original article [1], it was reported that there was a typo error in the article title. The word "fetal" should be "fatal".

Correct article title should read:

A case of afterload mismatch associated with shivering leading to fatal hypoxia in a COVID-19 patient

The original article has been updated.
